# Biological roles and clinical significance of estrogen and androgen receptors in head and neck cancers

**DOI:** 10.7150/jca.66707

**Published:** 2022-04-04

**Authors:** Chunhong Qin, Yan Lu, Huimin Zhang, Zhe Zhang, Wei Xu, Shuxin Wen, Wei Gao, Yongyan Wu

**Affiliations:** 1 Department of Biochemistry & Molecular Biology, Basic Medical College, Shanxi Medical University, Taiyuan 030001, Shanxi, China.; 2 Shanxi Key Laboratory of Otorhinolaryngology Head and Neck Cancer, Shanxi Province Clinical Medical Research Center for Precision Medicine of Head and Neck Cancer, Department of Otolaryngology Head & Neck Surgery, First Hospital of Shanxi Medical University, Taiyuan 030001, Shanxi, China.; 3 Department of Otolaryngology Head & Neck Surgery, The First Hospital, Jinzhou Medical University, Jinzhou 121001, Liaoning, China.; 4 Shanxi Eye Hospital, Taiyuan 030002, Shanxi, China.; 5 Department of Otolaryngology-Head and Neck Surgery, Shandong Provincial ENT Hospital, Cheeloo College of Medicine, Shandong University, Jinan 250022, Shandong, China.; 6 Department of Otolaryngology Head & Neck Surgery, Shanxi Bethune Hospital, Taiyuan 030032, Shanxi, China.; 7 General Hospital, Clinical Medical Academy, Shenzhen University, Shenzhen 518055, Guangdong, China.

**Keywords:** Androgen receptor, Estrogen receptor, Head and neck cancer, Molecular target, Biomarker, Cancer treatment

## Abstract

Head and neck cancers (HNC) include malignant tumors that grow in and around the mouth, larynx, throat, sinuses, nose, and salivary glands. Accumulating evidence in malignancies suggests the aberrant expressions of the estrogen receptor (ER) and the androgen receptor (AR) in HNC, such as in laryngeal cancer and cancer of the salivary gland. Moreover, the signaling pathways involving these receptors that mediate tumorigenesis, proliferation, apoptosis, migration, and invasion have been elucidated. This review summarizes the roles of ER and AR with the putative signaling pathways involved in HNC. We also discuss the potential application of ER- and AR-related therapies in HNC. However, most of the mechanisms underlying AR and ER involvement in the development of HNC remain elusive and warrant further studies. A comprehensive understanding of the functional roles and mechanisms of action of AR and ER in HNC will facilitate the development of better therapeutic strategies for this disease. Overall, studies on AR and ER provide a promising potential for the diagnosis and treatment of HNC in the future.

## Background

Head and neck cancers (HNC) are one of the most lethal and predominant tumors, worldwide, accounting for 5.7% of the total global mortality due to cancers 1-3. Its potential epidemiological trend shows an uneven regional distribution of disease burden. For instance, the Swedish Head and Neck Cancer Register (SweHNCR) report suggests that among the 9733 patients diagnosed with HNC between 2008 - 2015, approximately 10% died within six months of diagnosis 4. In 2019, an estimated 66,630 new cases of HNC were diagnosed and 14,620 individuals died due to this disease in the United States 5. Based on the epidemiological research data from 2008 to 2012, in Shanxi Province, China, the average annual incidence rate of laryngeal cancer was 0.70/10^5^ 6. Remarkably, the essential risk factors of HNC include tobacco and alcohol use, and infection by the human papillomavirus (HPV) 7, 8. Additionally, other factors have been identified by the epidemiological studies of HNC, including genetics, toxic exposure, diet, and environmental conditions 3, 9, 10.

Radiotherapy and chemotherapy are the most predominant adjuvant treatments for HNC 11. Radiotherapy can facilitate clinical outcomes to a certain extent but also is accompanied by some side effects of radiation, such as dry mouth symptoms and difficulties in chewing/swallowing 12, 13. Recently, the use of cetuximab and taxane has been on the rise 14, and the combined induction using gemcitabine and cisplatin suggests a new possibility for improving the recurrence-free survival rate 15. Despite these benefits, the disadvantages of chemoresistance, toxic side effects, and acute adverse events 16 have not been addressed, thus warranting further studies. In the past few years, due to the low toxicity of combination chemotherapy, that is, the integration of chemotherapy and combined androgen blockade (CAB) using the androgen receptor (AR) antagonist, the research attention for its clinical applicability has gained traction 17*.* For example, patients with advanced AR-positive salivary duct carcinoma exhibit increased overall survival after undergoing androgen-deprivation therapy (ADT) 18. Correspondingly, in breast cancer, the estrogen receptor-α66 (ERα66) serves as a molecular target for endocrine therapy and binds estrogen to mediate metastasis 19. Taken together, these studies indicate that AR and ER have promising roles in the management of cancers including HNC.

**Figure 1:**
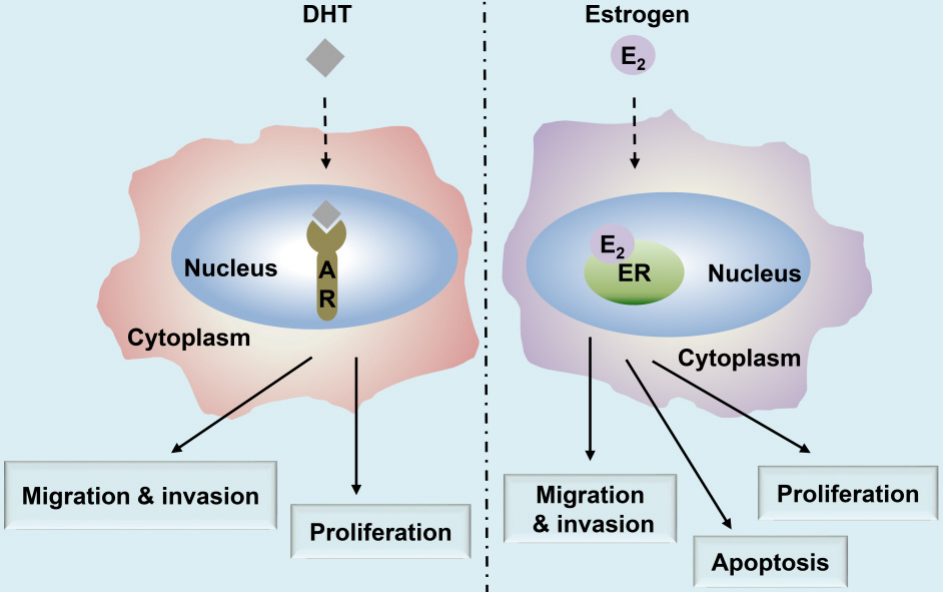
** Summary of the major functions of androgen and estrogen receptors in HNC.** The androgen receptor (AR)/estrogen receptor (ER)-mediated signaling pathways are activated upon binding with the corresponding ligands, androgen dihydrotestosterone (DHT) or estrogen (17β-estradiol, E_2_), respectively, resulting in the regulation of HNC behavior, including cell proliferation, apoptosis, migration, and invasion.

In this review, we have summarized the current knowledge on the functional roles of ER and AR in HNC (Fig. 1) and discussed their putative applications in treatment and diagnosis. Moreover, as anti-androgen and anti-estrogen therapies continue to be regarded as potential therapeutic strategies, targeting ER and AR signaling pathways may prove useful for the treatment of patients with HNC.

## ER-mediated signaling pathways

Estrogen receptors (ERs) are typical members belonging to the superfamily of nuclear receptors that include receptors involved in the action of steroid hormones, thyroid hormones, vitamin D, and several orphan receptors 20. These receptors are primarily classified into two categories, namely the classical nuclear receptors, comprising ERα and ERβ, localized in the nucleus and mediating the effects of estrogen and the membranous receptors, including the membranous components of the classical nuclear receptor and the G protein-coupled estrogen receptor 1 (GPER1) 21, 22.

**Figure 2:**
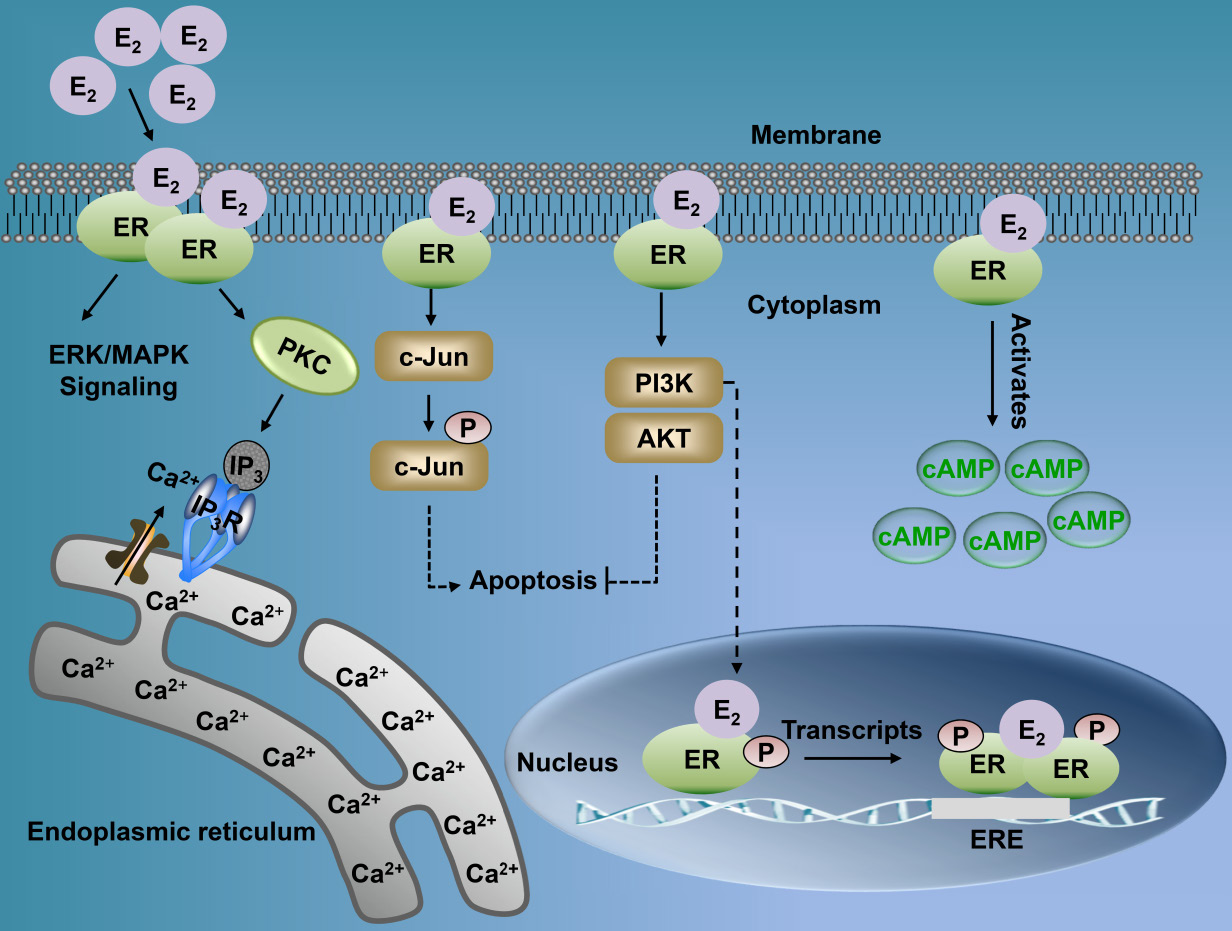
** Multiple signaling pathways of nuclear ER and membrane ER. Membrane-associated E_2_-ER signaling pathway.** (1) E_2_-ER activates ERK/MAPK signaling pathway; (2) The activation of PKC increases endogenous levels of Ca^2+^, which can induce tumorigenesis and metastasis; (3) E_2_-ER activates JNK pathway and eventually promotes apoptosis; (4) E_2_-ER involved in the PI3K/AKT pathway suppresses apoptosis; (5) E_2_-ER activates Camp; E_2_-ER signaling pathway in the nucleus. Phosphorylation of ER combined with E_2_ mediates the formation of ER-ERE complex, leading to transcription. ER: estrogen receptor; ERK: extracellular signal-regulated kinase; MAPK: mitogen-activated protein kinase; PKC: protein kinase C; IP_3_: inositol triphosphate; IP_3_R: inositol triphosphate receptor; cAMP: activation of adenylate cyclase; ERE: estrogen response element.

**Figure 3:**
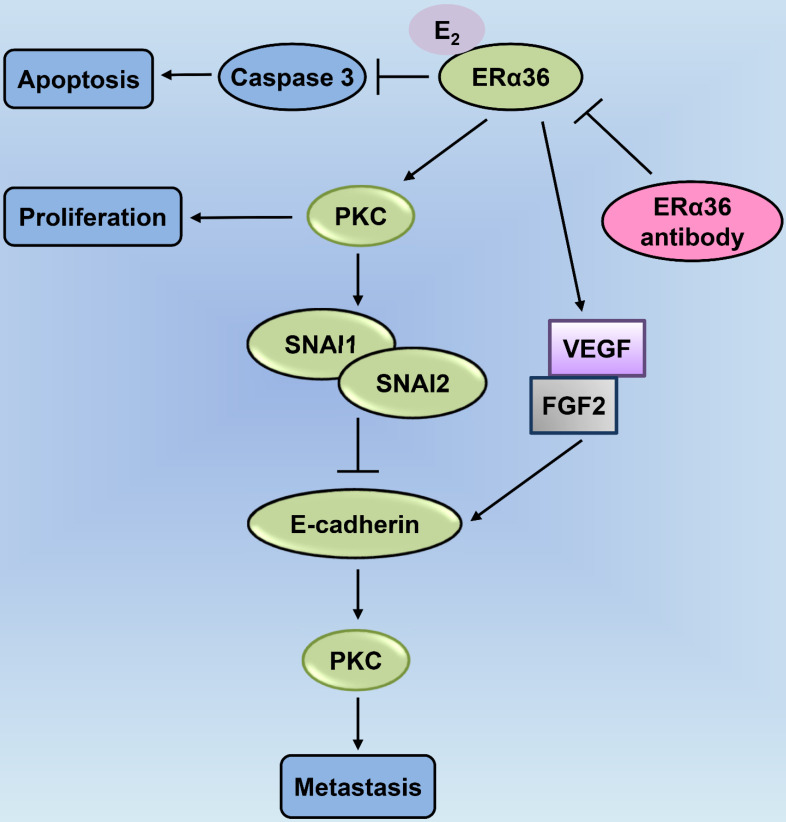
** PKC-mediated ERα36-E_2_ membrane signaling cascades are involved in cellular proliferation, anti-apoptosis, and metastasis in laryngeal cancer.** ERα36-associated E_2_ inhibits access of caspase-3 for promoting apoptosis; E_2_ activation of ERα36 enhances the levels of expression of vascular endothelial growth factor (VEGF) and fibroblast growth factor 2 (FGF2), blocked by the ERα36 antibody; ERα36-dependent E_2_ signaling increases PKC activity associated with cellular proliferation; ERα36 activates PKC through E_2_ that mediates the expression of the metastatic factors, SNAI1 and SNAI2; E_2_ enhances the expression of the metastatic factor, Snail, and downregulates E-cadherin (CDH1); ERα36 antibodies block both of these effects, leading to epithelial-to-mesenchymal transition (EMT) and enhanced metastasis. PKC: protein kinase C.

Estrogen either enters the cell or is synthesized within. It binds with the nuclear ER, thereby forming an ER homodimer or heterodimer 21. The activated ER interacts with the DNA enhancer estrogen response element (ERE) resulting in the ER-ERE complex, which further facilitates the formation of a transcription initiation complex that induces transcription, and thus, the ER exerts its functions 23. Estrogen can produce biological effects through the rapid activation of intracellular secondary signaling systems through the ER membrane 24. These effects include (1) the rapid activation of the extracellular signal-regulated kinase (ERK)/mitogen-activated protein kinase (MAPK) signaling pathway, (2) stimulation of adenylate cyclase (cAMP), thereby promoting the activity of cAMP-regulated gene transcription, and (3) activation of protein kinase C (PKC) increasing in endogenous Ca^2+^ 23-28 levels. Additionally, there are other ER-mediated pathways, including the c-Jun N-terminal kinase (JNK) pathway, which induces apoptosis, and the phosphatidylinositol 3 kinase (PI3K) pathway that inhibits apoptosis (Fig. 2) 22, 29, 30. Finally, the classical estrogen signaling is through the membrane-associated 17β-estradiol (E_2_)-ER binding, which triggers multiple rapid signal transduction cascades 24, 31 (Fig. 3).

## AR-mediated signaling pathways

**Figure 4:**
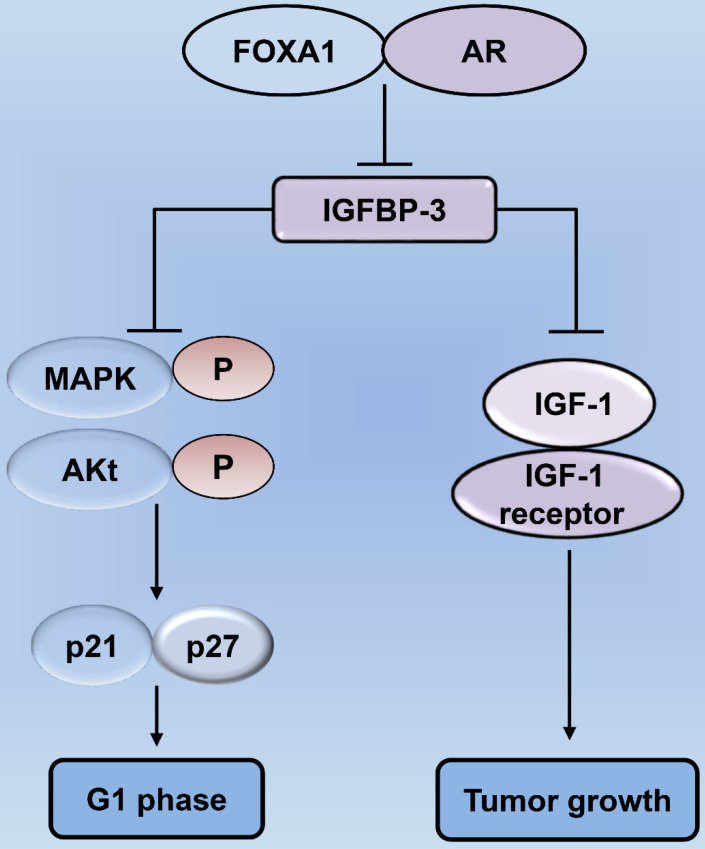
** Forkhead box protein A1 (FOXA1) regulates tumor cell proliferation by modulating the expression of insulin-like growth factor-binding protein 3 (IGFBP-3) in prostate cancer.** Decrease in the levels of FOXA1 and AR lead to an increase in IGFBP-3 expression, thereby inhibiting the proliferation of cancer cells. IGFBP-3 can suppress the biology of IGF-1 utilization, as well as inhibit the interaction between IGF-1 and the IGF-1 receptor, thereby exerting tumor-suppressive effects. Increased IGFBP-3 following FOXA1 depletion restrains the phosphorylation of signaling mediators, including MAPK and Akt, in the IGF-1 signaling pathway, thereby mediating cell cycle blockade in prostate cancer cells through p21 and p27. AR: androgen receptor; MAPK: mitogen-activated protein kinase.

The AR is similar to the ER in that they belong to the superfamily of nuclear receptors 32. The length of the AR gene, located on xq11-12, is greater than 90 kb. It has eight exons, wherein exon 1 is at the amino-terminus and is responsible for stimulating the gene transcription 33, 34. Thus, AR acts as a transcription factor and consequently translocates to the nucleus following the binding of the active androgen dihydrotestosterone (DHT) 33 (Fig. 1). The AR contains four functional regions as follows: the N-terminal domain, DNA-binding domain (DBD), ligand-binding domain, and a hinge 34. A typical signaling pathway involving AR consists of different stages 35. First, the AR binds to androgen (DHT) causing a conformational change, and thus, the AR separates from the heat shock proteins and enters the nucleus through the nuclear pore 34. In the nucleus, AR undergoes phosphorylation and dimerization and can recognize the androgen response elements (AREs) on target genes, thereby binds to them and causing chromatin remodeling in an effect to open up the regulatory regions of promoters, eventually recruiting co-activators for the formation of transcriptional complexes, ultimately leading to transcription of the target gene 33, 35. Forkhead box protein A1 (FOXA1) is a transcription factor of the AR target gene, which regulates its transcription as well as facilitates the interactions of AR with the chromatin 36. The contribution of AR and FOXA1 to the process of tumorigenesis 37 is illustrated in Fig. 4. Moreover, studies on salivary duct carcinoma show that treatment with DHT increases cell proliferation, migration, and invasion, while the AR inhibitor, fluorouracil, blocks the effects of DHT, suggesting that the androgen-AR signaling axis exerts effects on cellular proliferation, migration, and invasiveness 38.

## Functional roles of ER in the biological behavior of HNC

Recent studies show that the expression of ERα in laryngeal squamous cell carcinoma (LSCC) is higher relative to the corresponding normal tissues 39. A similar finding has been reported in malignant minor salivary gland tumors of the sinonasal tract, the majority of which are ER-positive 40. Likewise, the expression of ERβ is higher in head and neck squamous cell carcinoma (HNSCC) as compared to the normal epithelium 41. Besides, numerous reports confirm that ER immunoreactivity is present in approximately 50% - 80% of the cases in some HNC types 42-46. Taken together, these findings demonstrate that ERs are highly expressed in most HNC and are closely associated with its development. The roles and underlying mechanisms of action of ER in HNC progression are listed in Table 1.

Based on recent empirical evidence, ERs including ERα and ERβ, exert an anti-apoptotic effect in some HNC types 31, 47-49. In particular, in papillary thyroid cancer, ERα induces autophagy, which is a pro-survival catabolic phase owing to the stimulation of reactive oxygen species and extracellular signal-regulated kinases, wherein the inhibition of autophagy promotes apoptosis 47. Moreover, Shatalova et al. demonstrate high levels of ERβ expression in premalignant head and neck lesion cells MSK-Leuk1. Notably, exposure to E_2_ results in lower cellular apoptosis, and this estrogen-dependent apoptosis can be restored through supplementation with the anti-estrogen drug. However, the association of exposure to E2 with the inhibition of apoptosis and ERβ remains enigmatic and needs further evaluation 41. Although a group of researchers has provided considerable evidence suggesting that the activity of E_2_ is associated with its receptor ER, resulting in the enhanced proliferation and conferring anti-apoptotic potential to cancer cells according to their receptor profiles, the role of ER remains controversial. A plausible explanation could be due to the uneven expressions of different membrane-related ERs in laryngeal cancer cells 50.

ERα and ERβ exhibit differential expression patterns even in the same HNC type and may exert various effects. For instance, ERα and ERβ may play different functions in the tumor growth and progression of the medullary thyroid carcinoma (MTC) 51. Similarly, another study suggests that most cases of adenoid cystic carcinoma cases are positive for the expression of ERα and the five-year overall survival rate is significantly better in these cases relative to those with ERβ-positive expression 40. Furthermore, the differential expression patterns of ERα and ERβ may induce opposite proliferative effects on the same type of HNC. For instance, the expression of ERα is positive for the growth of papillary thyroid cancer (PTC) cells due to autophagy 47, whereas ERβ exerts an inhibitory effect 52. In thyroid carcinoma, ERs promote proliferation through different molecular mechanisms. Previous studies show that membrane-bound ERs mediate growth-promoting effects through classical genomic and non-genomic pathways, which are linked to the tyrosine kinase signaling cascades, including transduction through MAPK and PI3K 53. Another study demonstrates that 17beta-estradiol is an effective mitogen for benign and malignant thyroid tumor cells, as it can bind to the nuclear ERs, thereby promoting growth by activating the MAPK pathway 54. In addition, the ligand activation for GPR30 signaling, coupled with the upregulation of specific GPER genes, is involved in the proliferation of tumor cells, which implies that GPER can contribute to the tumorigenesis process 55, 56. Taken together, ERα and ERβ modulate the estrogen signaling at the genomic level, while the third membrane-bound estrogen receptor GPR30 is involved in non-genomic transduction mechanisms and occupies an equally important position in the development of HNC.

The expression of ERα66 is lower in aggressive LSCC, while a high ESR1 (a gene encoding ER) expression is associated with improved survival of patients with LSCC 57. Moreover, ERβ exhibits a significant role in the suppression of LSCC aggression, by reducing the aggressive epithelial-mesenchymal-transition (EMT) features including down-regulation of E-cadherin and concomitant activation of nuclear β-catenin 58. In contrast, ER expression is indicative of more aggressive behavior. Numerous studies have highlighted the crucial role of ERs in the progression of HNC due to enhanced invasion and migration through the E_2_ signaling 31, 59, 60. Combined ER and EGFR inhibition *in vitro* reportedly reduce HNSCC invasion but not proliferation as compared to both of them targeted singly. Moreover, the combined inhibition of EGF and estrogen signaling pathways can augment the inhibition of invasion relative to the blockade of each pathway separately 61.

## Effects of AR on the biological behavior of HNC

AR shows positive expression in HNCs, including oropharyngeal squamous cell carcinoma and salivary gland tumors. In a previous study, all six cases of salivary duct carcinoma and 14 cases of carcinoma ex pleomorphic adenoma (a subset of salivary gland carcinoma) showed strong nuclear immune reactivity for AR. Moreover, the uniform expression of AR in malignant salivary gland carcinoma has been demonstrated 62-66. In another study, AR expression was detected in tumor samples from oropharyngeal squamous cell carcinoma (OPSCC) patients. The results indicated that 16% (31/199) of the tumors were positive for AR expression, of which 61% (19/31) exhibited a strong expression 67. Tarakji et al. reported AR expression in 50% of the cases of pleomorphic salivary adenoma by immunohistochemical nuclear staining 68. Additionally, the high expression of AR is associated with oncogenesis. Wu et al. have investigated the oncogenic role of AR in oral squamous cell carcinoma (OSCC) and reported a high level of expression of AR in premalignant and malignant lesions relative to the normal mucosal tissues. They examined the clinical specimens and observed that OSCC cells expressed functional AR which promoted their growth 69. However, a few tumors show detectably lower rates 67, 70, 71 of 16% to 26% AR-positive expression. Despite the differential rates of AR expression, notably, the risk factors for malignant tumors are indispensable and deserve greater research attention. The potential functional roles of AR in HNC are listed in Table 2.

Higher expression of AR contributes to the proliferation of cells in different HNC types, including laryngeal carcinoma and juvenile nasopharyngeal fibroma. For instance, stimulation of AR by DHT in OSCC cells causes an increase in cyclin D1 expression, resulting in enhanced cell growth 69. Moreover, AR exerts a growth-promoting effect on cells in the larynx cancer 72, 73. In juvenile nasopharyngeal fibroma, the expression of AR is higher in tumor fibroblasts relative to the genital cells. Consequently, the growth rate of tumor fibroblasts increases due to the action of androgen, while that of the tumor cells is suppressed upon the addition of anti-androgen drugs 74. However, there is no definitive description of the mechanism underlying AR involvement in the proliferation of HNC, and little is known about how androgens impact proliferation upon binding to AR.

Interestingly, ARs exert opposite effects on proliferation in some cancer types. Recently, Hickey et al. have demonstrated that AR activation exhibits antitumor activity in ER-positive breast cancer. They found that the activation of AR altered the genomic distribution of ER and its important co-activators, leading to the repression of ER-regulated cell cycle genes and upregulation of AR-regulated tumor suppressors, thereby inhibiting proliferation of cancer cells 75. These opposing roles of AR may allow for the targeted development of new and better therapies for specific cancer types.

AR contributes to the migration of AR-positive OSCC cells 32. DHT acts as an AR-ligand that promotes AR-positive cell migration of OSCC by enhancing the expression of phosphorylated EGFR and AKT. Furthermore, upon treatment of SCC9 cells (AR-positive OSCC cells) with bicalutamide (an AR inhibitor), migration rate and the phosphorylation of EGFR and AKT, reduce. AR plays an important role in OSCC cell migration by regulating the EGFR signaling transduction 32. On the contrary, similar to ERs, ARs can also reduce invasion. The presence of an invasive micro-papillary component in salivary duct carcinoma that is AR-negative reportedly contributes to checking of the primary site; however, ordinary salivary duct carcinoma is positive for AR 76. In a study on small differentiated thyroid cancers (DTC), T1 cases according to the TNM Staging System, 2006 with AR-positive tumors showed more aggression as compared to the AR-negative tumors 77. Taking into account, the relatively scarce previous data from the literature, further studies are required to determine the aggressive role of AR expression in different types of HNCs.

## Clinical significance of ER and AR in HNC

According to their various biology functions, ERs play pivotal roles in the clinical disease progression of various HNC types. Accumulating evidence suggests that ERs are associated with shorter survival duration 78. On the one hand, higher ERα expression significantly influences survival 39. On the other hand, ERβ positive expression suggests improved survival rates in patients with oropharyngeal cancer 79. Notably, the relationship between ER expression and other factors exerts essential effects in some cancer types, as well as exhibits a great impact on the development of HNC. However, this influence depends on the type of cancer, along with the correlation between the factor and ER expression. Several studies confirm that ER is associated with prolactin receptor (PRLR) 39, epidermal growth factor receptor (EGFR) 61, and the apolipoprotein B mRNA-editing catalytic polypeptide 3 A (APOBEC3A, A3A) 80. The EGFR and ERα cross-talks are associated with poor prognoses because of enhanced tumor invasion 61. This implies that ER can interact with another factor, thereby influencing the development and progression of HNC. Considering all these findings based on previous studies, ER may serve as a novel predictor for HNC, particularly as a prognosticator or biomarker 55, 61.

The relationship between levels of AR and HNC clinical progression cannot be overlooked. In several cases, the role of AR in the progression of other cancer types is consistent with HNC, that is, favorable for tumor growth 69, 81. Empirical studies report that AR is closely associated with pathological classification, clinical stage, and lymph node metastasis, all of which can promote the growth of laryngeal carcinoma cells 73. Some reports emphasize that intraprostatic/tumor AR heterogeneity is associated with unfavorable clinical prognoses 82, while for laryngeal squamous cell carcinoma, no significant differences in the survival curves between the low and high expression groups 39 have been observed. Overall, the correlation between AR expression and prognosis in HNC has been rarely documented and warrants further examination.

## Application of ER and AR in HNC therapy

Increasing evidence demonstrates that ER and AR are involved in tumor initiation and progression. As described in previous sections, the expressions of ER and AR are high in HNC, indicating that they both are potential targets for HNC treatment. Some cases are amenable to anti-estrogen or anti-androgen therapy and are listed in Table 3 and Table 4, respectively.

Given the impact of ER, HNC treatment benefits from the advantageous effects of anti-estrogen drugs. Studies indicate growth inhibition of ER-positive OSCC cells upon treatment with an ER antagonist, tamoxifen. ER promotes the growth of OSCC cells, while tamoxifen suppresses cell growth 83. Nonetheless, the mechanisms through which these anti-estrogen agents exert anti-proliferative effects on laryngeal carcinoma cell lines remain unclear. However, the anti-estrogen agents prevent both translocation and nuclear binding of the receptor, thereby blocking ER, resulting in the inhibition of transcriptional activation of estrogen-responsive genes; they also possess potential disease-stabilizing effects in other cancer types 41, 84. Hence, we speculate that ER-positive patients may benefit from anti-estrogen treatment owing to the blockade of E_2_ and ER-related signaling cascades. This may underlie the effects of anti-estrogen treatment in HNC.

Furthermore, ER is associated with unfavorable outcomes after chemoradiation in patients with HNC. Tamoxifen is favorable for overcoming resistance to cisplatin in HNC-squamous cell carcinoma cell lines by inducing G1 arrest and sensitizing the cells to cisplatin-induced apoptosis 85. *In vitro* studies show that enhanced expression of human submaxillary gland androgen regulatory protein 3A (SMR3A) is accompanied by an upregulation in estrogen receptor 2 (ESR2) level after fractionated irradiation. ESR2-dependent regulation of SMR3A is supported by estradiol (E_2_) stimulation of SMR3A, and the co-treatment of estradiol (E_2_) with tamoxifen or fulvestrant, correspondingly, attenuate the regulation of SMR3A by ESR2. Both drugs significantly sensitize the HNC tumor cells to graded irradiation and accelerate apoptosis as evidenced by the results of the colony formation assay (CFA). These data suggest that ESR2 plays an important role in radioresistance 86. In thyroid cancer, the estrogen-related receptor γ (ERRγ, a nuclear receptor with high sequence identity to ERs) inverse agonist contributes to enhanced sensitivity towards radioiodine therapy, suggesting that it may be beneficial in restoring the unresponsiveness of poorly differentiated thyroid cancer cells to radioiodine therapy 87. On the contrary, ERα-positive oropharyngeal squamous carcinoma (OPSC) patients who underwent radiotherapy, show better overall, disease-free, progression-free, and relapse-free survival as compared to ERα-negative patients, suggesting that ERα may serve as a potential therapeutic target for OPSC 42. Taken together, the role of ER in HNC chemoradiation therapy remains controversial and needs further validation.

Given the high expression of AR in most HNC cases, patients undergo treatment through androgen deprivation therapy (ADT), using bicalutamide (an androgen receptor antagonist with a non-steroidal structure) 18, abiraterone 88, and flutamide 89. As first-line therapy for certain tumors, like the AR-positive salivary gland cancer 90, many patients benefit from ADT as it yields a higher response rate and better prognosis. Remarkably, 17 AR-positive salivary gland cancer patients who received ADT showed a 64.7% overall response rate, of which three showed a complete remission 91. Patients treated with first-line ADT exhibited a higher response rate of 45% (9/20) as compared with those who received first-line chemotherapy (14% (2/14)) 90. Additionally, relative to conventional chemotherapy, ADT has equivalent efficacy and less toxicity for unresectable locally advanced salivary gland carcinoma (SGC) 17. However, the emergence of ADT resistance has reduced its efficiency 92. The mechanisms underlying ADT resistance are linked to the low expression of androgen synthesis enzyme-encoding gene (SRD5A1) and low activity of the AR pathway. Thus, SRD5A1 may be a promising factor for enhancing responses to ADT in recurrent/metastatic SDC 92. Moreover, the benefits of ADT in HNC patients may depend on the level of AR expression.

Studies show that the combination of chemotherapy or radiotherapy with ADT is more effective for HNC treatment. In a case report, the ADT plus radiation regime was used to definitively treat androgen receptor-positive SDC. The patient showed a good clinical outcome with no clinical evidence of disease after 24 months post-treatment completion 93. In addition, AR positively correlates with overall survival in patients undergoing radiotherapy and chemotherapy after surgery for head and neck squamous cell carcinoma 94. Reports show that AR expression is an independent prognostic factor for overall survival and metastases-free survival 94, however, the relationship between AR expression and chemoradiation is ambiguous. In the future, clinical trials are expected to address whether combined androgen blockade is better than chemotherapy or radiotherapy alone as a first-line treatment for metastatic cancers, along with its correlation with AR.

## Conclusions and perspectives

This review summarized the potential roles of ER and AR in the progression of HNC. Based on the findings from several studies, further investigations are required to evaluate the functional roles and mechanisms underlying ER and AR involvement in the development of HNC and the putative benefits by alleviating negative effects on tumor growth and progression using antagonists of ER or AR. Differential levels of ER and AR elicit controversial effects in HNC progression, indicating a complexity in their status and roles, which requires further confirmation in certain cancer types.

FOXA1 and fatty acid synthase (FASN) may be associated with ADT resistance in prostate cancer 37, 95-99. Similarly, FOXA1 and FASN are also present in patients with AR-positive salivary gland cancer 100, and therefore, may be also associated with other HNC types. Targeting ERα36 can reduce the detrimental effects of E_2_ in laryngeal cancer, suggesting the importance of the identification of novel drugs that specifically target the ERα36 31. Overall, all findings indicate the importance of anti-estrogen and anti-androgen therapies or the production of novel drugs targeting AR and ER in HNC.

Going forward, the possible effects of ER and AR, such as their involvement in oncogenesis and mediating cellular proliferation, make them prime candidates of novel and potential biomarkers or/and targets for cancer diagnosis and treatment. Given that the levels of expression of AR or ER are regulated by their upstream signaling pathways or transcription factors, for instance, tamoxifen interrupts MAPK and PI3K signaling cascades to reduce E_2_-induced proliferation by decreasing ER expression 101, targeting the upstream pathways or transcription factors of ER and AR maybe a novel approach for the treatment of HNC.

## Figures and Tables

**Table 1:** The estrogen receptor (ER) expression, functions, and mechanisms in head and neck cancers (HNC)

Receptor	HNC Type	Expression	Prognosis	Function	Mechanism	Ref.
ER	Laryngeal cancer	Upregulation	Unfavorable	NA	NA	78
ERβ	Oropharyngeal cancer	Upregulation	Favorable	NA	NA	79
ERα	Laryngeal cancer	Upregulation	Unfavorable	NA	NA	39
ERα	HPV-positive oropharyngeal cancer	Upregulation	Favorable	NA	NA	80
ER	Laryngeal cancer	Upregulation	NA	Promote proliferation and inhibit apoptosis	NA	50
ERα	Papillary thyroid cancer (PTC)	Upregulation	NA	Promote growth of PTC and inhibit apoptosis	Activate ERK1/2 and autophagy	47
ERα36	Laryngeal cancer	Upregulation	NA	Inhibit apoptosis and increase aggression	Activate PKC and phospholipase D	31
ERβ	Laryngeal cancer	Upregulation	Unfavorable	Increase invasion	NA	102
ERβ	Tongue cancer	Upregulation	NA	Inhibit apoptosis	NA	41
ERβ	Tongue cancer	Upregulation	NA	Inhibit apoptosis and increase aggression	NA	48
ERβ1	Papillary thyroid cancer	Upregulation	NA	Inhibit proliferation	NA	52
ERα	Papillary thyroid cancer	Upregulation	NA	Promote growth and progression	NA	52
ER	Thyroid cancer	Upregulation	NA	Promote proliferation	Non-genomic pathways	101
ER	Thyroid carcinoma	Upregulation	NA	Promote growth of tumor cells	Genomic and non-genomic pathways	53
ER	Salivary gland cancer	Upregulation	Unfavorable	NA	NA	55
ERα66	Laryngeal cancer	Downregulation	Unfavorable	Increase aggression	NA	57
ERβ	Laryngeal cancer	Upregulation	NA	Inhibit aggression	Upregulate E-cadherin, activate β-catenin	58

Abbreviations: NA, not available; EGFR: growth factor receptor; ERK: extracellular signal-related kinase; Ref: reference.

**Table 2:** The androgen receptor (AR) expression, functions, and mechanisms in head and neck cancers (HNC)

HNC Type	Expression	Prognosis	Function	Mechanism	Ref.
Oral carcinoma	Upregulation	NA	Promote proliferation	Upregulate cyclin D1 level and promote cell growth	69
Juvenile nasopharyngeal fibroma	Upregulation	NA	Promote proliferation	NA	74
Laryngeal cancer	Upregulation	NA	Promote proliferation	NA	73
Salivary duct cancer	Upregulation	NA	Increase invasion	NA	76
Micro-papillary salivary duct cancer	Downregulation	NA	Increase aggression	NA	76
Laryngeal cancer	Downregulation	Unfavorable	Increase invasion	NA	102
Differentiated thyroid cancer	Upregulation	NA	Increase aggression	NA	77

Abbreviations: NA, not available; Ref: reference.

**Table 3:** Application of anti-estrogen therapy in HNC

Receptor	ER level	HNC Type	Treatment	Response	Outcome	Ref.
ER	Positive	Laryngeal cancer	Tamoxifen citrate	Suppress the growth of cells	NA	103
ERβ	Positive	Tongue cancer	Fulvestrant	Promote apoptosis	NA	41
ER	Positive	Salivary gland cancer	Tamoxifen/tore-mifene	Negative ER status after therapy	Long-term stability of disease	84

Abbreviations: NA, not available; Ref: reference.

**Table 4:** Application of anti-androgen therapy in HNC

HNC Types	AR level	Treatment	Response	Outcome	Ref.
Salivary gland cancer	Positive	Bicalutamide and leuprorelin acetate	Clinical benefit rate (75%)	Favorable prognosis	17
Salivary duct carcinoma	Positive	Bicalutamide/combination with Goserelin (LHRH analog)	Stable disease for 32% patients and 50% patients with clinical benefit	Favorable prognosis	18
Salivary duct carcinoma	Positive	Bicalutamide with external beam radiotherapy	NA	Favorable prognosis	93
Parotid gland adenocarcinoma	Positive	Bicalutamide and Triptorelin (LHRH analog)	A complete remission	Favorable prognosis	104
Salivary gland cancer	Positive	Cyproaterone acetate and triptorelin	Overall response rate 64.7%	Favorable prognosis	91
Salivary Duct Carcinoma	Positive	Leuprolide and bicalutamide	Good partial response after 3 and 6 months	Favorable prognosis	105

Abbreviations: NA, not available; Ref: reference; LHRH: luteinizing hormone-releasing hormone.

## References

[1] Patterson RH, Fischman VG, Wasserman I (2020). Global Burden of Head and Neck Cancer: Economic Consequences, Health, and the Role of Surgery. Otolaryngol Head Neck Surg.

[2] Siegel RL, Miller KD, Jemal A (2020). Cancer statistics, 2020. CA Cancer J Clin.

[3] Holt J, Walter V, Yin X (2021). Integrative Analysis of miRNAs Identifies Clinically Relevant Epithelial and Stromal Subtypes of Head and Neck Squamous Cell Carcinoma. Clin Cancer Res.

[4] Talani C, Mäkitie A, Beran M, Holmberg E, Laurell G, Farnebo L (2019). Early mortality after diagnosis of cancer of the head and neck - A population-based nationwide study. PLoS One.

[5] Siegel RL, Miller KD, Jemal A (2019). Cancer statistics, 2019. CA Cancer J Clin.

[6] Qi H, Chen W, Zhang C (2021). Epidemiological Analysis of 1234 Cases of Laryngeal Cancer in Shanxi Province, China. Cancer Control.

[7] Rettig EM, D'Souza G (2015). Epidemiology of head and neck cancer. Surg Oncol Clin N Am.

[8] Yang DL, Shi Y, Tang YM (2019). Effect of HPV Infection on the Occurrence and Development of Laryngeal Cancer: A Review. J Cancer.

[9] Cohen N, Fedewa S, Chen AY (2018). Epidemiology and Demographics of the Head and Neck Cancer Population. Oral Maxillofac Surg Clin North Am.

[10] Vučičević Boras V, Fučić A, Baranović S (2019). Environmental and behavioural head and neck cancer risk factors. Cent Eur J Public Health.

[11] Petit C, Lacas B, Pignon JP (2021). Chemotherapy and radiotherapy in locally advanced head and neck cancer: an individual patient data network meta-analysis. Lancet Oncol.

[12] Weppler S, Quon H, Schinkel C, Yarschenko A, Barbera L, Harjai N (2021). Patient-Reported Outcomes-Guided Adaptive Radiation Therapy for Head and Neck Cancer. Front Oncol.

[13] Brook I (2020). Late side effects of radiation treatment for head and neck cancer. Radiat Oncol J.

[14] Schlichting JA, Pagedar NA, Chioreso C, Lynch CF, Charlton ME (2019). Treatment trends in head and neck cancer: Surveillance, Epidemiology, and End Results (SEER) Patterns of Care analysis. Cancer Causes Control.

[15] Zhang Y, Chen L, Hu GQ (2019). Gemcitabine and Cisplatin Induction Chemotherapy in Nasopharyngeal Carcinoma. N Engl J Med.

[16] Hsieh MC, Wang CC, Yang CC, Lien CF, Wang CC, Shih YC (2021). Tegafur-Uracil versus 5-Fluorouracil in Combination with Cisplatin and Cetuximab in Elderly Patients with Recurrent or Metastatic Head and Neck Squamous Cell Carcinoma: A Propensity Score Matching Analysis. Biology (Basel).

[17] Fushimi C, Tada Y, Takahashi H (2018). A prospective phase II study of combined androgen blockade in patients with androgen receptor-positive metastatic or locally advanced unresectable salivary gland carcinoma. Ann Oncol.

[18] Boon E, van Boxtel W, Buter J (2018). Androgen deprivation therapy for androgen receptor-positive advanced salivary duct carcinoma: A nationwide case series of 35 patients in The Netherlands. Head Neck.

[19] Wang Q, Jiang J, Ying G (2018). Tamoxifen enhances stemness and promotes metastasis of ERα36+ breast cancer by upregulating ALDH1A1 in cancer cells. Cell Res.

[20] Pakdel F (2018). Molecular Pathways of Estrogen Receptor Action. Int J Mol Sci.

[21] Safe S, Kim K (2008). Non-classical genomic estrogen receptor (ER)/specificity protein and ER/activating protein-1 signaling pathways. J Mol Endocrinol.

[22] Fuentes N, Silveyra P (2019). Estrogen receptor signaling mechanisms. Adv Protein Chem Struct Biol.

[23] Marino M, Galluzzo P, Ascenzi P (2006). Estrogen signaling multiple pathways to impact gene transcription. Curr Genomics.

[24] Prossnitz ER, Arterburn JB (2015). International Union of Basic and Clinical Pharmacology. XCVII. G Protein-Coupled Estrogen Receptor and Its Pharmacologic Modulators. Pharmacol Rev.

[25] Arnal JF, Lenfant F, Metivier R (2017). Membrane and Nuclear Estrogen Receptor Alpha Actions: From Tissue Specificity to Medical Implications. Physiol Rev.

[26] Pietras RJ, Márquez-Garbán DC (2007). Membrane-associated estrogen receptor signaling pathways in human cancers. Clin Cancer Res.

[27] Niwa T, Takanobu J, Suzuki K, Sato Y, Yamaguchi Y, Hayashi SI (2020). Characterization of a membrane-associated estrogen receptor in breast cancer cells and its contribution to hormone therapy resistance using a novel selective ligand. J Steroid Biochem Mol Biol.

[28] Verma A, Schwartz N, Cohen DJ, Boyan BD, Schwartz Z (2019). Estrogen signaling and estrogen receptors as prognostic indicators in laryngeal cancer. Steroids.

[29] Van Tine BA, Crowder RJ, Ellis MJ (2011). ER and PI3K independently modulate endocrine resistance in ER-positive breast cancer. Cancer Discov.

[30] Bosch A, Li Z, Bergamaschi A (2015). PI3K inhibition results in enhanced estrogen receptor function and dependence in hormone receptor-positive breast cancer. Sci Transl Med.

[31] Schwartz N, Chaudhri RA, Hadadi A, Schwartz Z, Boyan BD (2014). 17Beta-estradiol promotes aggressive laryngeal cancer through membrane-associated estrogen receptor-alpha 36. Horm Cancer.

[32] Liu X, Qing S, Che K, Li L, Liao X (2019). Androgen receptor promotes oral squamous cell carcinoma cell migration by increasing EGFR phosphorylation. Onco Targets Ther.

[33] Culig Z, Santer FR (2014). Androgen receptor signaling in prostate cancer. Cancer Metastasis Rev.

[34] Li P, Chen J, Miyamoto H (2017). Androgen Receptor Signaling in Bladder Cancer. Cancers (Basel).

[35] Yeoh CC, Dabab N, Rigby E (2018). Androgen receptor in salivary gland carcinoma: A review of an old marker as a possible new target. J Oral Pathol Med.

[36] Augello MA, Hickey TE, Knudsen KE (2011). FOXA1: master of steroid receptor function in cancer. EMBO J.

[37] Imamura Y, Sakamoto S, Endo T (2012). FOXA1 promotes tumor progression in prostate cancer via the insulin-like growth factor binding protein 3 pathway. PLoS One.

[38] Kamata YU, Sumida T, Murase R, Nakano H, Yamada T, Mori Y (2016). Blockade of Androgen-induced Malignant Phenotypes by Flutamide Administration in Human Salivary Duct Carcinoma Cells. Anticancer Res.

[39] Fei MJ, Xu YN, Wang JD (2018). The expression and clinical progression of androgen, estrogen and prolactin receptor in laryngeal squamous cell carcinoma. Lin Chung Er Bi Yan Hou Tou Jing Wai Ke Za Zhi.

[40] Luo SD, Su CY, Chuang HC, Huang CC, Chen CM, Chien CY (2009). Estrogen receptor overexpression in malignant minor salivary gland tumors of the sinonasal tract. Otolaryngol Head Neck Surg.

[41] Shatalova EG, Klein-Szanto AJ, Devarajan K, Cukierman E, Clapper ML (2011). Estrogen and cytochrome P450 1B1 contribute to both early- and late-stage head and neck carcinogenesis. Cancer Prev Res (Phila).

[42] Koenigs MB, Lefranc-Torres A, Bonilla-Velez J, Patel KB, Hayes DN, Glomski K (2019). Association of Estrogen Receptor Alpha Expression With Survival in Oropharyngeal Cancer Following Chemoradiation Therapy. J Natl Cancer Inst.

[43] Liang L, Williams MD, Bell D (2019). Expression of PTEN, Androgen Receptor, HER2/neu, Cytokeratin 5/6, Estrogen Receptor-Beta, HMGA2, and PLAG1 in Salivary Duct Carcinoma. Head Neck Pathol.

[44] Vannucchi G, De Leo S, Perrino M (2015). Impact of estrogen and progesterone receptor expression on the clinical and molecular features of papillary thyroid cancer. Eur J Endocrinol.

[45] Inoue H, Oshimo K, Miki H (1993). Immunohistochemical study of estrogen receptor and estradiol on papillary thyroid carcinoma in young patients. J Surg Oncol.

[46] Xue L, Yan H, Chen Y (2019). EZH2 upregulation by ERα induces proliferation and migration of papillary thyroid carcinoma. BMC Cancer.

[47] Fan D, Liu SY, van Hasselt CA (2015). Estrogen receptor α induces prosurvival autophagy in papillary thyroid cancer via stimulating reactive oxygen species and extracellular signal regulated kinases. J Clin Endocrinol Metab.

[48] Ishida H, Wada K, Masuda T (2007). Critical role of estrogen receptor on anoikis and invasion of squamous cell carcinoma. Cancer Sci.

[49] Font-Díaz J, Jiménez-Panizo A, Caelles C (2020). Nuclear receptors: Lipid and hormone sensors with essential roles in the control of cancer development. Semin Cancer Biol.

[50] Schwartz N, Verma A, Muktipaty C, Bivens C, Schwartz Z, Boyan BD (2019). Estradiol receptor profile and estrogen responsiveness in laryngeal cancer and clinical outcomes. Steroids.

[51] Cho MA, Lee MK, Nam KH (2007). Expression and role of estrogen receptor alpha and beta in medullary thyroid carcinoma: different roles in cancer growth and apoptosis. J Endocrinol.

[52] Huang Y, Dong W, Li J, Zhang H, Shan Z, Teng W (2014). Differential expression patterns and clinical significance of estrogen receptor-α and β in papillary thyroid carcinoma. BMC Cancer.

[53] Derwahl M, Nicula D (2014). Estrogen and its role in thyroid cancer. Endocr Relat Cancer.

[54] Manole D, Schildknecht B, Gosnell B, Adams E, Derwahl M (2001). Estrogen promotes growth of human thyroid tumor cells by different molecular mechanisms. J Clin Endocrinol Metab.

[55] Aquino G, Collina F, Sabatino R (2018). Sex Hormone Receptors in Benign and Malignant Salivary Gland Tumors: Prognostic and Predictive Role. Int J Mol Sci.

[56] Chen Z, Xuan Q, Zhao D, Wu S, Yin J, Pan H (2020). Roles of G protein-coupled receptor 30 in the effects of genistein on apoptosis and cell cycle in human thyroid squamous cells SW579. Wei Sheng Yan Jiu.

[57] Verma A, Schwartz N, Cohen DJ (2020). Loss of Estrogen Receptors is Associated with Increased Tumor Aggression in Laryngeal Squamous Cell Carcinoma. Sci Rep.

[58] Goulioumis AK, Fuxe J, Varakis J, Repanti M, Goumas P, Papadaki H (2009). Estrogen receptor-beta expression in human laryngeal carcinoma: correlation with the expression of epithelial-mesenchymal transition specific biomarkers. Oncol Rep.

[59] Sumida T, Ishikawa A, Mori Y (2016). Stimulation of the Estrogen Axis Induces Epithelial-Mesenchymal Transition in Human Salivary Cancer cells. Cancer Genomics Proteomics.

[60] Wen DS, Wu JZ, Fu SM, Liu B, Li XX, Dong JJ (2005). Effects of 17 beta-estradiol on the adhesion, invasion and motility potential of salivary mucoepidermoid carcinoma Mc3 cells. Zhonghua Kou Qiang Yi Xue Za Zhi.

[61] Egloff AM, Rothstein ME, Seethala R, Siegfried JM, Grandis JR, Stabile LP (2009). Cross-talk between estrogen receptor and epidermal growth factor receptor in head and neck squamous cell carcinoma. Clin Cancer Res.

[62] Nasser SM, Faquin WC, Dayal Y (2003). Expression of androgen, estrogen, and progesterone receptors in salivary gland tumors. Frequent expression of androgen receptor in a subset of malignant salivary gland tumors. Am J Clin Pathol.

[63] Uijen M, Lassche G, van Engen-van Grunsven A (2020). Systemic therapy in the management of recurrent or metastatic salivary duct carcinoma: A systematic review. Cancer Treat Rev.

[64] Schvartsman G, Bell D, Rubin ML (2021). The tumor immune contexture of salivary duct carcinoma. Head Neck.

[65] Mitani Y, Lin SH, Pytynia KB, Ferrarotto R, El-Naggar AK (2020). Reciprocal and Autonomous Glucocorticoid and Androgen Receptor Activation in Salivary Duct Carcinoma. Clin Cancer Res.

[66] Hardy N, Thompson J, Mehra R, Drachenberg CB, Hatten K, Papadimitriou JC (2020). Parotid Salivary Duct Carcinoma With a Prominent Squamous Component: Immunohistochemical Profile, Diagnostic Pitfalls, and Therapeutic Implications. Int J Surg Pathol.

[67] Mohamed H, Aro K, Jouhi L (2018). Expression of hormone receptors in oropharyngeal squamous cell carcinoma. Eur Arch Otorhinolaryngol.

[68] Tarakji B, Kujan O (2011). An immunohistochemical study of androgen receptor in carcinoma arising in pleomorphic salivary adenoma. Med Oral Patol Oral Cir Bucal.

[69] Wu TF, Luo FJ, Chang YL (2015). The oncogenic role of androgen receptors in promoting the growth of oral squamous cell carcinoma cells. Oral Dis.

[70] Marocchio LS, Giudice F, Corrêa L, Pinto Junior Ddos S, de Sousa SO (2013). Oestrogens and androgen receptors in oral squamous cell carcinoma. Acta Odontol Scand.

[71] Jo U, Song JS, Choi SH, Nam SY, Kim SY, Cho KJ (2020). Primary squamous cell carcinoma of the salivary gland: immunohistochemical analysis and comparison with metastatic squamous cell carcinoma. J Pathol Transl Med.

[72] Chen B, Wang J, Li W, Ji W (2006). Expression of androgen receptor and estrogen receptor in carcinoma of larynx. Lin Chuang Er Bi Yan Hou Ke Za Zhi.

[73] Wang JQ, Dong Z, Zhu JG, Bu GX, Guo XF (1991). Androgen receptors in laryngeal carcinoma and their clinical significance. Chin Med J (Engl).

[74] Hagen R, Romalo G, Schwab B, Hoppe F, Schweikert HU (1994). Juvenile nasopharyngeal fibroma: androgen receptors and their significance for tumor growth. Laryngoscope.

[75] Hickey TE, Selth LA, Chia KM, Laven-Law G, Milioli HH, Roden D (2021). The androgen receptor is a tumor suppressor in estrogen receptor-positive breast cancer. Nat Med.

[76] Yamamoto H, Uryu H, Segawa Y, Tsuneyoshi M (2008). Aggressive invasive micropapillary salivary duct carcinoma of the parotid gland. Pathol Int.

[77] Magri F, Capelli V, Rotondi M (2012). Expression of estrogen and androgen receptors in differentiated thyroid cancer: an additional criterion to assess the patient's risk. Endocr Relat Cancer.

[78] Lukits J, Remenár E, Rásó E, Ladányi A, Kásler M, Tímár J (2007). Molecular identification, expression and prognostic role of estrogen- and progesterone receptors in head and neck cancer. Int J Oncol.

[79] Grsic K, Opacic IL, Sitic S, Milkovic Perisa M, Suton P, Sarcevic B (2016). The prognostic significance of estrogen receptor β in head and neck squamous cell carcinoma. Oncol Lett.

[80] Kano M, Kondo S, Wakisaka N (2019). Expression of estrogen receptor alpha is associated with pathogenesis and prognosis of human papillomavirus-positive oropharyngeal cancer. Int J Cancer.

[81] Li TH, Zhao H, Peng Y, Beliakoff J, Brooks JD, Sun Z (2007). A promoting role of androgen receptor in androgen-sensitive and -insensitive prostate cancer cells. Nucleic Acids Res.

[82] Grivas PD, Robins DM, Hussain M (2013). Predicting response to hormonal therapy and survival in men with hormone sensitive metastatic prostate cancer. Crit Rev Oncol Hematol.

[83] Chang YL, Hsu YK, Wu TF (2014). Regulation of estrogen receptor α function in oral squamous cell carcinoma cells by FAK signaling. Endocr Relat Cancer.

[84] Elkin AD, Jacobs CD (2008). Tamoxifen for salivary gland adenoid cystic carcinoma: report of two cases. J Cancer Res Clin Oncol.

[85] Tavassoli M, Soltaninia J, Rudnicka J, Mashanyare D, Johnson N, Gäken J (2002). Tamoxifen inhibits the growth of head and neck cancer cells and sensitizes these cells to cisplatin induced-apoptosis: role of TGF-beta1. Carcinogenesis.

[86] Rong C, ÉFRC M, Hess J (2018). Estrogen Receptor Signaling in Radiotherapy: From Molecular Mechanisms to Clinical Studies. Int J Mol Sci.

[87] Kim J, Hwang H, Yoon H, Lee JE, Oh JM, An H (2020). An orally available inverse agonist of estrogen-related receptor gamma showed expanded efficacy for the radioiodine therapy of poorly differentiated thyroid cancer. Eur J Med Chem.

[88] Locati LD, Perrone F, Cortelazzi B, Imbimbo M, Bossi P, Potepan P (2014). Activity of abiraterone in rechallenging two AR-expressing salivary gland adenocarcinomas, resistant to androgen-deprivation therapy. Cancer Biol Ther.

[89] Jaspers HC, Verbist BM, Schoffelen R, Mattijssen V, Slootweg PJ, van der Graaf WT (2011). Androgen receptor-positive salivary duct carcinoma: a disease entity with promising new treatment options. J Clin Oncol.

[90] Viscuse PV, Price KA, Garcia JJ, Schembri-Wismayer DJ, Chintakuntlawar AV (2019). First Line Androgen Deprivation Therapy vs. Chemotherapy for Patients With Androgen Receptor Positive Recurrent or Metastatic Salivary Gland Carcinoma-A Retrospective Study. Front Oncol.

[91] Locati LD, Perrone F, Cortelazzi B (2016). Clinical activity of androgen deprivation therapy in patients with metastatic/relapsed androgen receptor-positive salivary gland cancers. Head Neck.

[92] van Boxtel W, Verhaegh GW, van Engen-van Grunsven IA, van Strijp D, Kroeze LI, Ligtenberg MJ (2020). Prediction of clinical benefit from androgen deprivation therapy in salivary duct carcinoma patients. Int J Cancer.

[93] Soper MS, Iganej S, Thompson LD (2014). Definitive treatment of androgen receptor-positive salivary duct carcinoma with androgen deprivation therapy and external beam radiotherapy. Head Neck.

[94] Rades D, Seibold ND, Schild SE, Bruchhage KL, Gebhard MP, Noack F (2013). Androgen receptor expression: prognostic value in locally advanced squamous cell carcinoma of the head and neck. Strahlenther Onkol.

[95] Chou FJ, Chen Y, Chen D, Niu Y, Li G, Keng P (2019). Preclinical study using androgen receptor (AR) degradation enhancer to increase radiotherapy efficacy via targeting radiation-increased AR to better suppress prostate cancer progression. EBioMedicine.

[96] Teng M, Zhou S, Cai C, Lupien M, He HH (2021). Pioneer of prostate cancer: past, present and the future of FOXA1. Protein Cell.

[97] Zadra G, Ribeiro CF, Chetta P, Ho Y, Cacciatore S, Gao X (2019). Inhibition of de novo lipogenesis targets androgen receptor signaling in castration-resistant prostate cancer. Proc Natl Acad Sci U S A.

[98] Wen S, Niu Y, Lee SO, Yeh S, Shang Z, Gao H (2016). Targeting fatty acid synthase with ASC-J9 suppresses proliferation and invasion of prostate cancer cells. Mol Carcinog.

[99] Kregel S, Bagamasbad P, He S, LaPensee E, Raji Y, Brogley M (2020). Differential modulation of the androgen receptor for prostate cancer therapy depends on the DNA response element. Nucleic Acids Res.

[100] Dalin MG, Watson PA, Ho AL, Morris LG (2017). Androgen Receptor Signaling in Salivary Gland Cancer. Cancers (Basel).

[101] Kumar A, Klinge CM, Goldstein RE (2010). Estradiol-induced proliferation of papillary and follicular thyroid cancer cells is mediated by estrogen receptors alpha and beta. Int J Oncol.

[102] Atef A, El-Rashidy MA, Elzayat S, Kabel AM (2019). The prognostic value of sex hormone receptors expression in laryngeal carcinoma. Tissue Cell.

[103] Robbins KT, Vu TP, Diaz A, Varki NM (1994). Growth effects of tamoxifen and estradiol on laryngeal carcinoma cell lines. Arch Otolaryngol Head Neck Surg.

[104] Locati LD, Quattrone P, Bossi P, Marchianò AV, Cantù G, Licitra L (2003). A complete remission with androgen-deprivation therapy in a recurrent androgen receptor-expressing adenocarcinoma of the parotid gland. Ann Oncol.

[105] Jeong I, Moyers J, Thung I, Thinn MM (2020). Combination Chemohormonal Therapy in Metastatic Salivary Duct Carcinoma. Am J Case Rep.

